# NSUN2 affects diabetic retinopathy progression by regulating MUC1 expression through RNA m^5^C methylation

**DOI:** 10.1186/s12967-024-05287-4

**Published:** 2024-05-19

**Authors:** Runze Wang, Wei Xue, Feifei Kan, Huiying Zhang, Di Wang, Lei Wang, Jianwen Wang

**Affiliations:** 1 Eye Hospital, The First Affiliated Hospital of Harbin Medical University, Harbin, 150000 China; 2 Department of Urology, Shengjing Hospital of China Medical University, Shenyang, 110004 China

**Keywords:** Diabetic retinopathy, NSUN2, RNA m^5^C methylation, MUC1, ALYREF

## Abstract

**Background:**

Diabetic retinopathy (DR) is the leading cause of blinding eye disease among working adults and is primarily attributed to the excessive proliferation of microvessels, which leads to vitreous hemorrhage and retinal traction, thereby significantly impairing patient vision. NSUN2-mediated RNA m^5^C methylation is implicated in various diseases, and in this investigation, we focused on elucidating the impact of NSUN2 on the regulation of the expression of the downstream gene MUC1, specifically through RNA m^5^C methylation, on the progression of DR.

**Method:**

Utilizing Microarray analysis, we examined patient vitreous fluid to pinpoint potential therapeutic targets for DR. Differential expression of NSUN2 was validated through qRT-PCR, Western blot, and immunofluorescence in human tissue, animal tissue, and cell model of DR. The relationship between NSUN2 and DR was explored in vitro and in vivo through gene knockdown and overexpression. Various techniques, such as MeRIP-qPCR and dot blot, were applied to reveal the downstream targets and mechanism of action of NSUN2.

**Results:**

The levels of both NSUN2 and RNA m^5^C methylation were significantly elevated in the DR model. Knockdown of NSUN2 mitigated DR lesion formation both in vitro and in vivo. Mechanistically, NSUN2 promoted MUC1 expression by binding to the RNA m^5^C reader ALYREF. Knockdown of ALYREF resulted in DR lesion alterations similar to those observed with NSUN2 knockdown. Moreover, MUC1 overexpression successfully reversed a series of DR alterations induced by NSUN2 silencing.

**Conclusions:**

NSUN2 regulates the expression of MUC1 through ALYREF-mediated RNA m^5^C methylation, thereby regulating the progression of DR and providing a new option for the treatment of DR in the future.

**Supplementary Information:**

The online version contains supplementary material available at 10.1186/s12967-024-05287-4.

## Introduction

It is estimated that the global prevalence of diabetes will more than double from the current level by 2050, which will also lead to an increase in the number of patients suffering from diabetic microvascular complications. DR is emerging as the number one blinding eye disease in the working population [1, 2]. The main features of this disease are that under the stimulation of persistent hyperglycemia, the retinal vascular permeability of diabetic patients increases, retinal microvascular endothelial cells become dysfunctional, their proliferative capacity is enhanced, and the secretion of angiogenic mediators increases, leading to the massive generation of microvessels [3]. Studies have shown that DR progression is associated with abnormal migration, proliferation, and microvessel formation in human retinal microvascular endothelial cells (HRMECs) [4]. Angiogenesis implies the transition of DR from early non-proliferative DR to late proliferative DR, which is an important marker of disease progression. This pathological vascular growth is uncontrolled by the organism. In DR, the integrity of the endothelium and vasculature is damaged, leading to irregular vascular structure and disturbed blood flow, resulting in vitreous hemosiderosis and traction retinal detachment, which can lead to severe vision loss [5]. Therefore, restoring endothelial cell function and mitigating and eliminating retinal neovascularisation are important goals in the management of patients with DR [2].

Like DNA methylations, RNA is also modified by a variety of chemical modifications, such as N^6^-methyladenosine (m^6^A), N^1^-methyladenosine (m^1^A), N^7^-methyladenosine (m^7^G), and 5-methylcytosine (m^5^C) [6]. To date, more than 160 chemical modifications have been discovered in RNA. These modifications can affect the function and metabolic process of RNA and are considered important regulators of gene expression [7]. Therefore, RNA methylation has become an important and rapidly developing research topic in biomedical research [8]. M^5^C is a common RNA modification that is widely present in rRNAs, tRNAs, mRNAs, and noncoding RNAs [4, 9]. The m^5^C modification of eukaryotic RNA is catalyzed by the NSUN family of proteins (NSUN1, NSUN2, NSUN3, NSUN4, NSUN5, NSUN6, and NSUN7) and DNA Methyltransferase homolog 2 [10]. The m^5^C modification regulates RNA export and translation and maintains RNA stability [11–13] and thus has important regulatory effects on biological processes such as cell proliferation, differentiation, aging, and migration [14, 15].

Among all RNA m^5^C regulatory proteins, the RNA m^5^C regulatory protein NSUN2 has been studied most intensively [16]. NSUN2 is a major mRNA methyltransferase located mainly in the nucleus and catalyzes the formation of 5-methylcytosine in RNA [17, 18]. The human NSUN2 gene is located at chromosomal region 5p15.3 [19]; it contains two separate domains, the N-terminaldomain (NTD), which has methyltransferase activity, and the extended C-terminaldomain (CTD). The NTD functions as a methyltransferase, and its catalytic activity can be enhanced by the CTD. The CTD has a low affinity for RNA and cannot bind to RNA by itself, which may be related to the promotion of the formation of efficient enzyme-substrate complexes [20]. Research shows that NSUN2 is related to RNA m^5^C methylation in a variety of eye diseases. In corneal epithelial wound healing (CEWH), NSUN2-mediated m^5^C modification of UHRF1 mRNA can regulate CEWH. Knockdown of NSUN2 significantly delayed the CEWH process in vivo and inhibited the proliferation and migration of human corneal epithelial cells in vitro [21]. In retinoblastoma (RB), the NSUN2/ALYREF/m^5^C-PFAS carcinogenic cascade is an important trigger of RB and can promote purine synthesis during the pathological process. NSUN2 methylates PFAS transcripts and promotes their RNA stability in an RNA m^5^C reader ALYREF-dependent manner [22].

The aim of this study was to explore the regulatory role of NSUN2-mediated m^5^C modification in the pathogenesis of DR and reveal its potential mechanism, which can better delay the progression of DR and provide new inspiration for the clinical discovery of new DR treatments.

## Materials and methods

### Human tissue collection

A total of 50 patients participated in the study, and all signed an informed consent form. The perfusion was ensured to be closed during vitreous fluid collection, the tube screw cap of the vitreous cutter was unscrewed, a 5 mL syringe was connected, and the syringe was slowly pumped outward through the 23 G minimally invasive vitreous cutting system. After collection of the vitreous fluid, the fluid in the syringe was immediately passed through a cryostat, placed in liquid nitrogen, quickly transferred to a -80 °C freezer and stored. After excluding unavailable samples and appropriate statistical analysis, we selected 20 pairs of samples for subsequent testing.

### Microarray analysis

Total RNA was extracted using TRIzol (Invitrogen, USA), purified using an RNeasy Mini Kit (Qiagen, Germany) and checked for RINs by an Agilent Bioanalyzer 2100 (Santa Clara, CA, US) to determine RNA integration. Groups of RNA samples were then hybridized to slides, and the slides were scanned on an Agilent microarray scanner (Santa Clara, CA, US). The data were extracted using the Feature Extraction software 12.0.3.1 (Santa Clara, CA, US). The raw data were normalized using the quantile algorithm. Differential genes were screened using the p value of the t test, which was based on a p value of ≤ 0.05 and a multiplicity of differences ≥ 2.

### Cell culture

HRMECs were cultured in endothelial cell medium (SCIENCECELL, USA). The cells were divided into two groups: one group was cultured in endothelial cell medium supplemented with 5.5 mmol/L glucose, and the other group was cultured in endothelial cell medium supplemented with 30 mmol/L glucose.

### Cell transfection

NSUN2 siRNA, ALYREF siRNA, and the MUC1 overexpression plasmid were purchased from GenePharma (Suzhou, China). The sequences are shown in Additional file 1:Table S1. HRMECs were transfected using Lipofectamine(lipo) 2000 transfection reagent (Invitrogen, USA). Before transfection, the medium was changed to serum-free medium without double antibody, and then, a mixture of Lipo2000 and siRNA was added. After 4–6 h, the medium was changed to medium with a glucose concentration of 30 mmol/L. The culture was continued for 48 h, after which the cells were collected for the next experiment.

### Quantitative real-time PCR (qRT-PCR)

RNA was extracted from tissues and cells using TRIzol (Invitrogen, USA). The RNA was reverse-transcribed into cDNA by a reverse transcription kit (Sevenbio, Beijing, China). cDNA was subjected to qRT-PCR and quantified with SYBR Green Master Mix (Roche, Beijing, China). The data were analyzed by the 2^−ΔΔCt^ method. The primer sequences are shown in in Additional file 1:Table S2.

### Western blot

Tissues and cells were washed in precooled PBS. RIPA lysis buffer (Beyotime Biotechnology, Shanghai, China) and protease inhibitor were added to extract the proteins. Equal amounts of protein were added to SDS‒PAGE gels for separation, transferred to PVDF membranes, blocked with 5% skim milk for 1 h, and then incubated with the corresponding primary antibodies at 4 °C overnight. The details of the antibodies used are shown in in Additional file 1:Table S3. The next day, the PVDF membrane was washed three times with TBST, incubated with the secondary antibody at room temperature for 2 h, washed three additional times, and visualized via enhanced chemiluminescence (ECL) staining.

### Cell counting Kit-8 (CCK-8) assay

Cell proliferation ability was determined by a CCK8 assay. The cells were added to 96-well plates at 1 × 10^3^ cells/well. CCK8 reagent (Sevenbio, Beijing, China) was added at 24 h, 48 h and 72 h, and the optical density (OD) was measured at a wavelength of 450 nm.

### 5-Ethynyl-2′deoxyuridine (EdU) assay

4 × 10^3^ cells were added to the wells of six-well plates. After the culture conditions were completed, the cells were stained with EdU according to the manufacturer’s protocol. EdU reagent (10 μm; Beyotime Biotechnology, Shanghai, China) was added to each well, and the cells were incubated at 37 °C for 2 h. The cells were stained with azide 488 (green) and Hoechst 33,342 (blue). Afterward, fluorescence detection was performed, and the cell proliferation rate was calculated by the ratio of EdU-positive cells (green fluorescence) to Hoechst 33,342-positive cells (blue fluorescence).

### Wound healing assay

When the cell density reached 90%, we drew a straight line close to the bottom of the six-well plate with a 200 µL yellow pipette. The cell debris was removed using PBS. Observations and photographs were recorded at 0 h and 24 h.

### Transwell assay

The upper chamber of the Transwell was filled with 200 µL of cell suspension resuspended in serum-free medium, and 500 µL of medium containing 10% FBS was added to the lower chamber. After incubating at 37 °C for 24 h, the chamber was taken out, fixed with 4% paraformaldehyde for 30 min, and stained with crystal violet for 30 min, after which the cell status was observed and photographed under a microscope.

### Tube formation assay

50 µL of Matrigel (Corning, USA) was added to each well of a 96-well plate and incubated at 37 °C for 1 h to allow solidification. After 2 × 10^5^ cells were added to each well, the 96-well plate was placed back into a 37 °C incubator, and the cell status was observed and photographed under a microscope after 4–6 h.

### Dot-blot assay

Total RNA was extracted with TRIzol reagent (Invitrogen, USA), diluted to different concentrations, denatured at high temperature, cooled and transferred to a nylon film. After UV cross-linking, the cells were blocked with 5% skim milk and incubated with a m^5^C antibody (ab214727, Abcam) overnight at 4 °C. The next day, the membrane was washed three times with TBST, and the membrane was incubated with an anti-rabbit IgG antibody (SA00001-2, Proteintech) for 1.5 h at room temperature. After being washed three times with TBST, the membrane was visualized by ECL development.

### Methylated RNA immunoprecipitation-PCR (MeRIP-qPCR) analysis

Total RNA was extracted with TRIzol. M^5^C (Mab-006-500, Diagenode) or IgG (11201D, Invitrogen) antibody was added, and they were incubated at 4 °C overnight. The next day, magnetic beads were added and shaken overnight at 4 °C. RNA was eluted from the magnetic beads, and relative gene expression was detected via qPCR.

### Construction of mouse models

30 DB/M and 30 DB/DB mice were divided into two groups randomly, and there are 15 DB/M mice and 15 DB/DB mice in each group. One group is treated as early-stage DR group and the other is treated as late-stage DR groups. When the mice in early-stage DR group were 10 weeks and in late-stage DR groups were 24 weeks old, the mice were euthanized, and their eyeballs were collected for sample preparation. Additionally, 60 DB/DB mice were randomly divided into two groups of 30 mice each and were intraperitoneally injected with 2.2.2-tribromoethanol (Aladdin, USA) (0.1 mL/10 g). After the pupils were dilated with mydriatic eye drops, a 5-µL syringe with a 33-gauge needle (Hamilton, USA) was vertically inserted into the vitreous cavity behind the corneoscleral limbus (1.5 mm), and the mixture was slowly injected with 1.5 µL of AAV (Hanbio Tech, Shanghai, China). Finally, antibiotic eye ointment was applied to the eyes to prevent infection. After the mice were raised for the corresponding period, the mice were euthanized, and the eyeballs were completely removed and stored in PBS or 4% paraformaldehyde for subsequent experiments.

### Immunofluorescence staining

Mouse eyeballs were embedded in paraffin, sectioned, dehydrated, dewaxed, and incubated with 10% serum for 30 min. The serum was discarded, and primary and secondary antibody working solutions were added to the sections for incubation. Finally, the cell nuclei were stained with 4,6-diamino-2-phenylindole (DAPI) (Sevenbio, Beijing, China) and observed under a fluorescence microscope.

### Fluorescein fundus angiography (FFA)

After the mice were anesthetized, mydriatic eye drops were used to fully dilate the pupils. Gatifloxacin Eye Gel (China) was applied to the eyes to keep them moist. After intraperitoneal injection of sodium fluorescein (Beijing, China), the retinal blood vessels were observed under a digital fundus camera (Canon, Japan). The presence of blurred vessel boundaries and diffuse blurred fluorescence were regarded as fluorescein leakage.

### Hematoxylin and eosin (HE) staining of retinas

Mouse retinal tissues were fixed in 4% paraformaldehyde and made into paraffin sections. After dewaxing, the sections were stained with hematoxylin staining solution and eosin staining solution, dehydrated with ethanol, and mounted for observation.

### Statistical analysis

All the data were analyzed using GraphPad software (version 7.0), and the results are expressed as the means ± standard deviations (SD). The Kolmogorov-Smirov test was first used to figure out whether the data conforms to normal distribution. For the data conforms to normal distribution, the differences between two groups were analyzed using the independent t-test, and the differences between multiple groups were compared using one-way analysis of variance (ANOVA). For the data that does not fit normal distribution, the Mann-Whitney U test was used to analyze the differences between two groups, and the Kruskal-Wallis test was used to analyze the differences between multiple groups. All the experiments were repeated at least three times using different tissue samples and different batches of cells. P values less than 0.05 were considered to indicate statistical significance.

## Results

### NSUN2 expression and RNA m^5^C methylation levels are elevated in DR

**Fig. 1:**
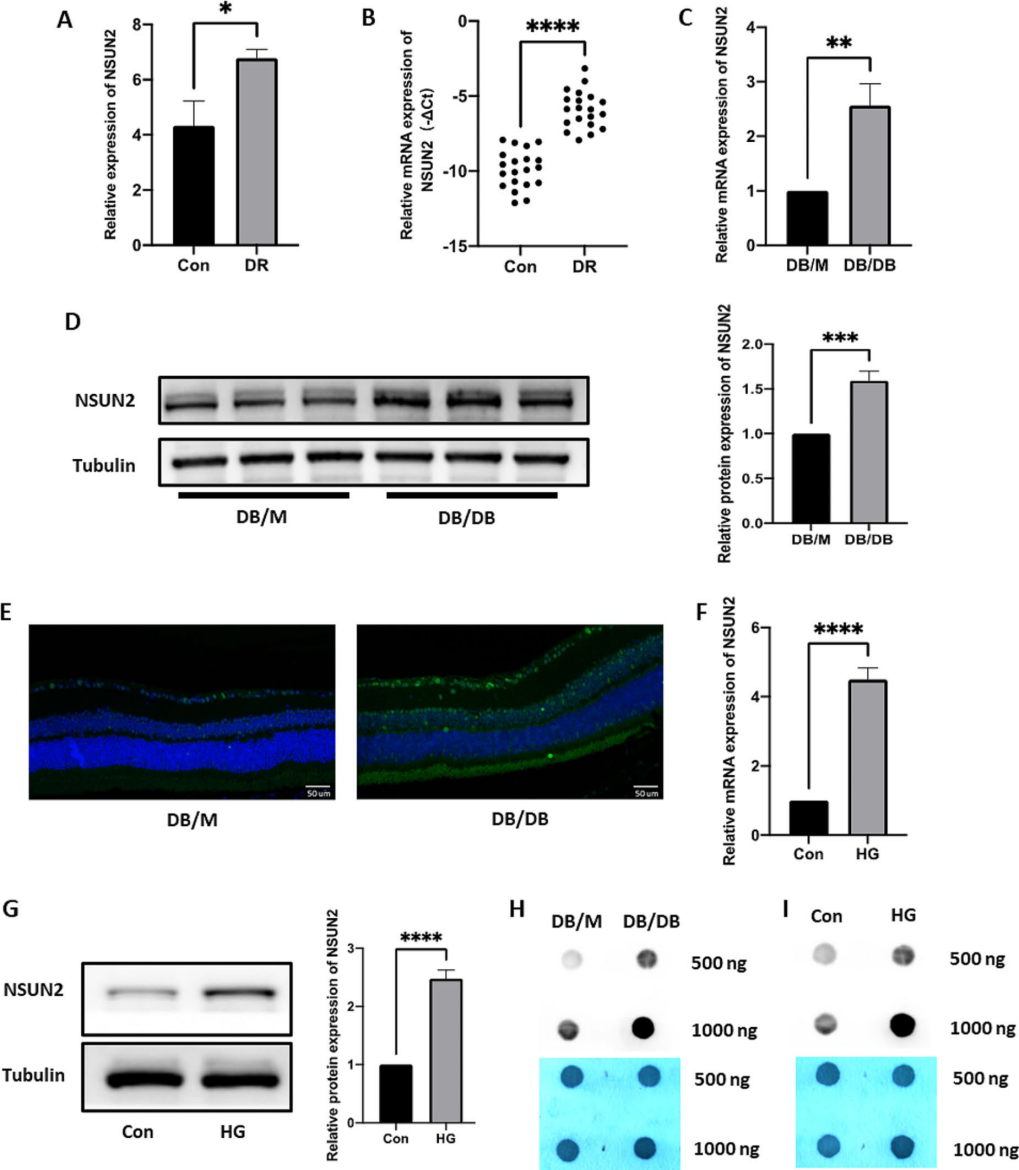
NSUN2 and RNA m^5^C methylation level is highly expressed in DR. (**A**) The target gene NSUN2 was significantly differentially expressed according to the Microarray analysis. (**B**) NSUN2 mRNA expression in DR and non-DR patient samples. (**C**) NSUN2 mRNA expression in DB/M versus DB/DB mice. (**D**) NSUN2 protein expression in DB/M versus DB/DB mice. (**E**) Differential immunofluorescence staining for NSUN2 in DB/M and DB/DB mice. (**F**) Differential expression of NSUN2 mRNA at the cellular level. (**G**) Differential expression of the NSUN2 protein at the cellular level. (**H**) Differences in RNA m^5^C levels in DB/M and DB/DB mice. **I** Differences in RNA m^5^C at the cellular level

To systematically identify new targets involved in DR, vitreous fluid was collected from patients for Microarray analysis, and a total of 140 upregulated genes and 259 downregulated genes were identified (Additional file 1: Fig. S1A-C). According to the Microarray analysis results, NSUN2 was significantly upregulated in the vitreous fluid of DR patients (Fig. 1A). Among the other up and down-regulated genes, no product has been found can be interact with NSUN2 in vivo according to exist studies [23–28]. The qRT-PCR results were consistent with the Microarray analysis results (Fig. 1B). Subsequently, control mice (DB/M), early-stage DR mice and late-stage DR mice (DB/DB) were cultured. The retinal tissue of the mice was peeled off, and the protein and RNA were extracted for verification of NSUN2 expression. In early-stage DR mice, no differential expression of NSUN2 can be observed (Additional file 1: Fig. S2A). In DB/DB mice, the results indicated that the mRNA and protein expression levels were both significantly upregulated, which is consistent with previous conclusions (Fig. 1C, D). In addition, the IHC results also indicated that the expression of NSUN2 in the DB/DB group was significantly greater than that in the DB/M group (Fig. 1E). HRMECs were cultured in high-glucose medium to establish a DR cell model. Both qRT‒PCR and Western blot confirmed that, compared with those in the control group, the mRNA and protein expression levels of NSUN2 in the DR cell model group were significantly higher (Fig. 1F, G). To verify whether DR induces RNA m^5^C methylation, a dot blot was used to assess the level of RNA m^5^C methylation. The results showed that m^5^C was highly modified in DB/DB mice and in the DR cell model group (Fig. 1H, I). In summary, these results indicate that the RNA m^5^C methylation level is elevated in DR, that there is high expression of NSUN2, and that NSUN2 may affect the progression of DR through the RNA m^5^C methylation pathway.

### NSUN2 enhances DR RNA m^5^C methylation levels, proliferation and angiogenesis in vitro

**Fig. 2:**
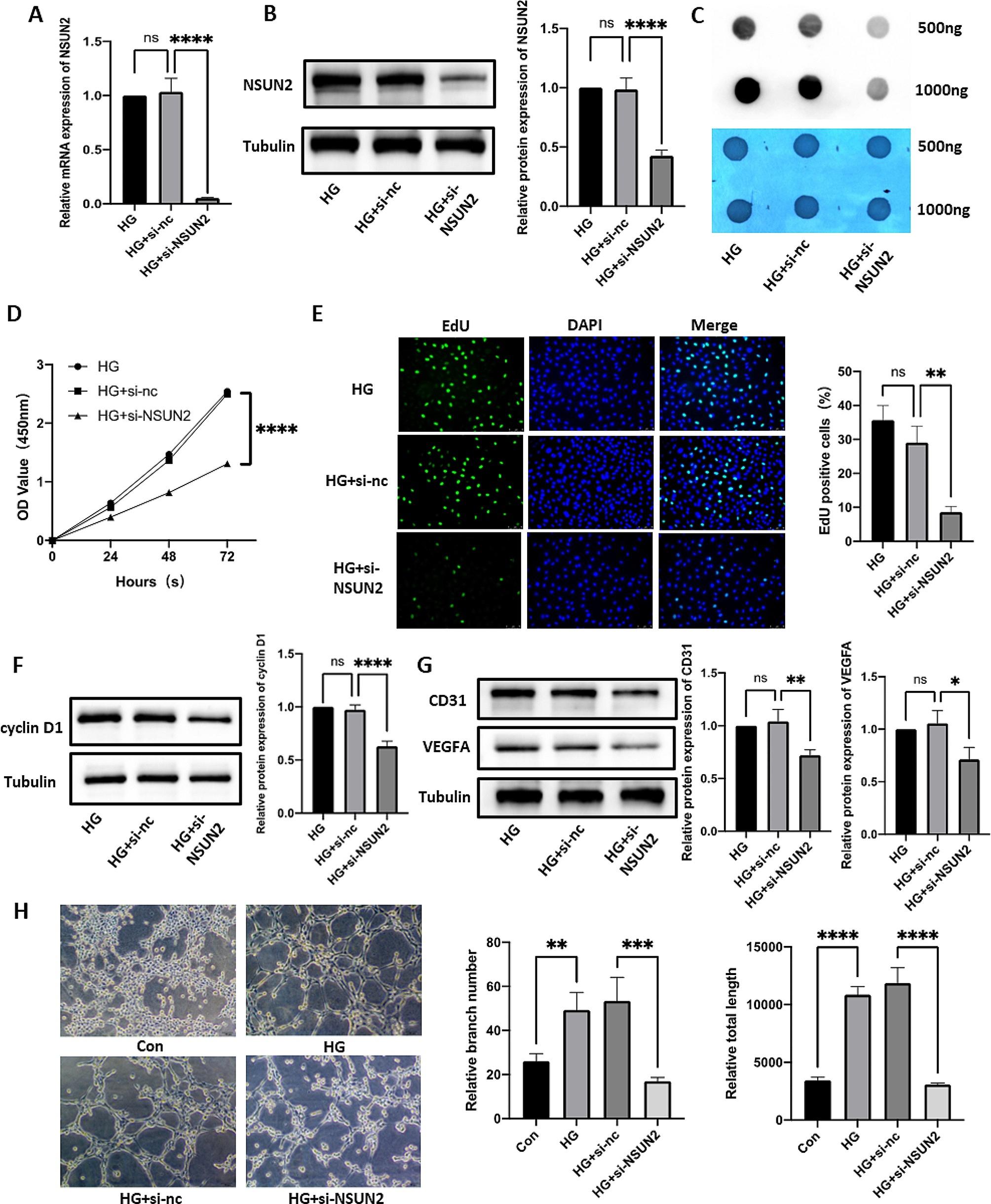
Effect of NSUN2 on cell proliferation and angiogenesis. (**A**) qRT-PCR was used to evaluate the transfection efficiency of NSUN2. (**B**) Western blot analysis of the transfection efficiency of NSUN2. (**C**) Changes in the RNA m^5^C methylation level after si-NSUN2 treatment. (**D**) A CCK-8 assay was used to measure the effect of si-NSUN2 on cell proliferation. (**E**) An EdU assay was used to measure the effect of si-NSUN2 on cell proliferation. (**F**) Western blot analysis of the changes in cyclin D1 protein levels after si-NSUN2 transfection. (**G**) Western blot analysis of changes in CD31 and VEGFA protein levels after si-NSUN2 transfection. (**H**) The effects of HG and si-NSUN2 on the tube formation ability of HRMECs were measured via a tube formation assay

We constructed a siRNA targeting NSUN2 and verified by qRT-PCR and Western blot that it successfully downregulated the expression of NSUN2 in HRMECs (Fig. 2A, B). Changes in RNA m^5^C methylation levels were detected via a dot blot assay. The results showed that, compared with the control, the knockdown of NSUN2 resulted in a significant reduction in RNA m^5^C methylation (Fig. 2C). Cell proliferation was detected by CCK8 and EdU assays. CCK8 results showed that knockdown of NSUN2 resulted in a significant decrease in cell proliferation (Fig. 2D). EdU assays also confirmed this result. The percentage of EdU-positive cells was significantly reduced after NSUN2 knockdown, and the proliferative ability of the cells was significantly decreased (Fig. 2E). In addition, after transfection with si-NSUN2, the expression level of the proliferation-related protein cyclin D1 was significantly reduced (Fig. 2F). In summary, these results confirmed that NSUN2 enhances DR vascular proliferation and RNA m^5^C methylation levels in vitro. Angiogenesis plays an important role in the progression of DR. Therefore, we next explored whether NSUN2 can affect angiogenesis in DR. In the tube formation assay, the tube formation ability of the si-NSUN2 group was significantly lower than that of the control group (Fig. 2H). As classic indicators of angiogenesis, the expression levels of CD31 and VEGFA were significantly downregulated in the si-NSUN2 group (Fig. 2G), which once again verified that NSUN2 can enhance the angiogenesis ability of HRMECs at the protein level.

### Knockdown of NSUN2 significantly inhibits DR migration in vitro

**Fig. 3:**
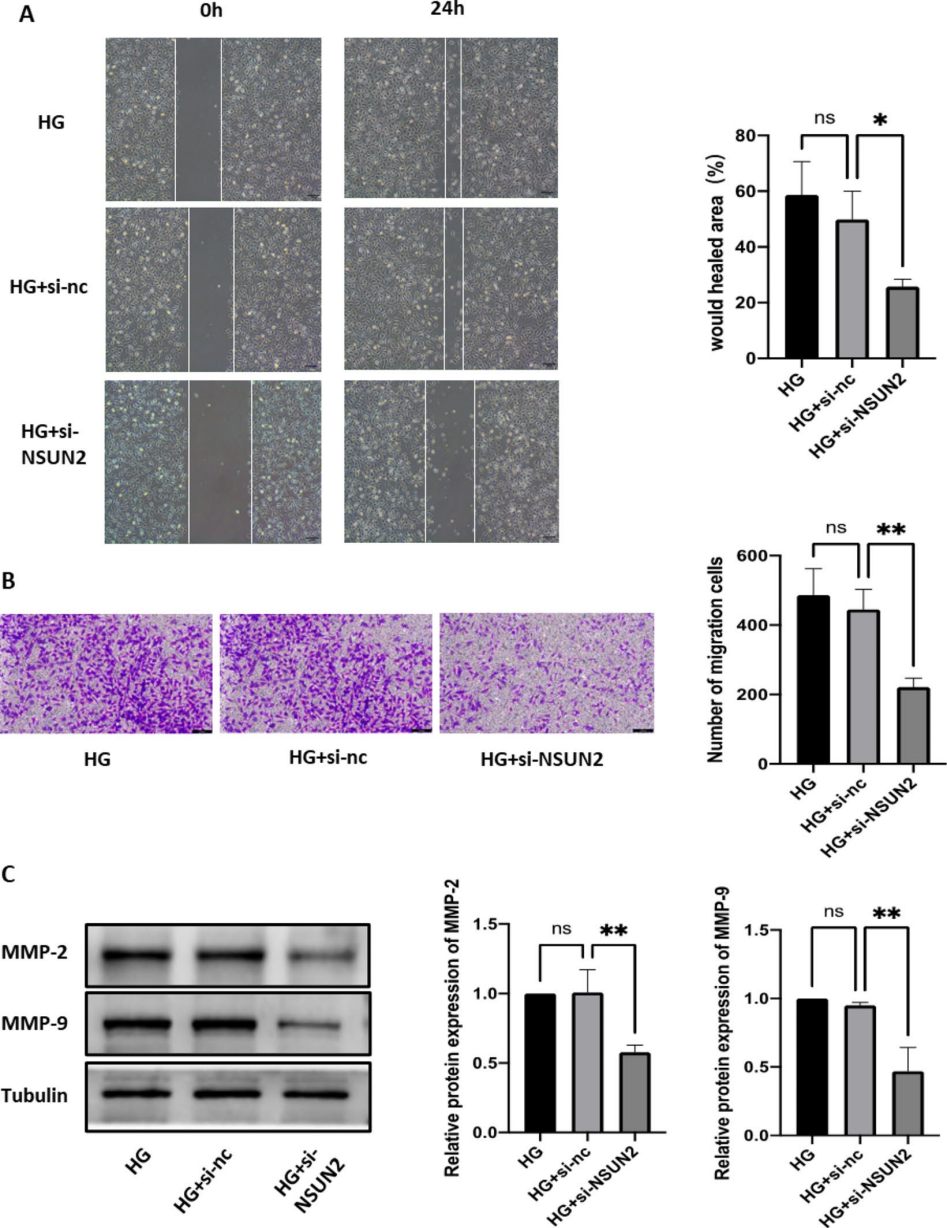
Effect of NSUN2 on cell migration. (**A**) Wound healing assay was performed to detect changes in cell migration ability after si-NSUN2 treatment. (**B**) Transwell assays were used to measure the alteration in cell migration ability after si-NSUN2 transfection. (**C**) Western blot analysis of the changes in the MMP-2 and MMP-9 protein levels after si-NSUN2 treatment

We further explored whether NSUN2 can affect DR vessel migration. First, a wound healing assay was performed. 24 h after scratching, the scratch healing ability of the si-NSUN2 group was significantly lower than that of the control group (Fig. 3A), confirming that NSUN2 can enhance the migration ability of HRMECs. Next, a Transwell experiment was performed. In the si-NSUN2 group, the number of cells that migrated to the lower chamber was significantly lower than that in the control group (Fig. 3B). Among the migration-related indicators, the expression of MMP-2 and MMP-9 was significantly downregulated in the si-NSUN2 group, which confirmed the ability of NSUN2 to enhance the migration of HRMECs at the protein level (Fig. 3C). Based on these results, we concluded that NSUN2 can significantly enhance the migration of HRMECs in DR.

### MUC1 is a potential RNA m^5^C methylation downstream regulator of NSUN2

**Fig. 4:**
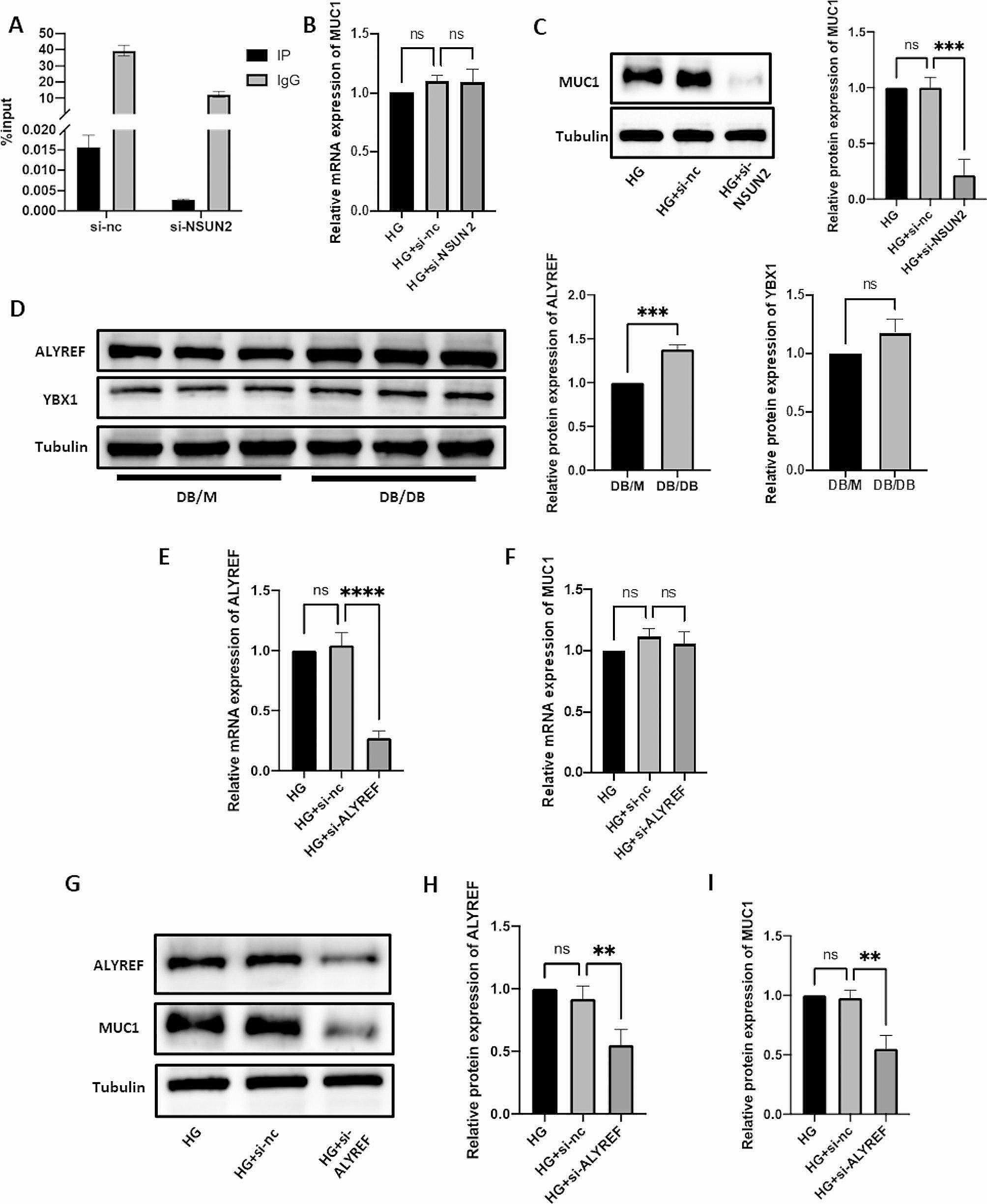
MUC1 is a downstream target of NSUN2 in DR and is regulated by ALYREF. (**A**) The reduction in the MUC1 m^5^C modification level after NSUN2 knockdown was examined by MeRIP-qPCR. (**B**) qRT-PCR was used to evaluate changes in MUC1 mRNA levels after si-NSUN2 treatment. (**C**) Western blot analysis of changes in MUC1 protein levels after si-NSUN2 treatment. (**D**) Western blot analysis of the expression of ALYREF and YBX1 in DB/M and DB/DB mice. (**E, G, H**) qRT-PCR and western blot were used to evaluate the efficiency of ALYREF knockdown. (**F, G, I**) qRT‒PCR and western blot were used to evaluate the changes in MUC1 after si-ALYREF

First, based on a literature review, 77 molecules that may be regulated by NSUN2 were screened out [21]. Second, NSUN2-mediated RNA m^5^C methylation can promote mRNA translation. Therefore, because the RNA m^5^C level of NSUN2 is significantly increased in DR, we screened 39 upregulated genes from 77 molecules that may be regulated by NSUN2 (proteins whose expression level increased by at least 1.5 times were classified as upregulated proteins) (Additional file 1: Table S4). Third, after further screening among the 39 upregulated molecules, MUC1 attracted our interest. Studies have shown that MUC1 can promote hypoxia-driven angiogenesis by regulating multiple proangiogenic factors [29]. This mechanism is similar to the pathogenesis of DR, so we conducted a MeRIP-qPCR experiment. The MeRIP-qPCR results showed that knocking out NSUN2 significantly reduced the enrichment of RNA m^5^C methylation in MUC1 (Fig. 4A). Subsequently, the expression levels of MUC1 mRNA and protein were verified in HRMECs with NSUN2 knockdown. Although the MUC1 mRNA expression did not change significantly (Fig. 4B), the MUC1 protein expression was significantly downregulated (Fig. 4C). The above results indicate that MUC1 is a potential RNA m^5^C methylation downstream regulator of NSUN2.

### The RNA m^5^C reader ALYREF is an important recognition protein of RNA m^5^C-methylated MUC1 mRNA

Studies have shown that RNA m^5^C methylation can be directly recognized by readers (ALYREF or YBX1), affecting RNA processing and subsequent effects [30]. In our experiments, we found that the protein expression of ALYREF was elevated in the retinal tissues of DB/DB mice, whereas the change in the protein expression of YBX1 was not significant (Fig. 4D). Therefore, we next focused on whether ALYREF is involved in the regulation of MUC1 expression and affects the progression of DR. We constructed an ALYREF siRNA and verified the successful knockdown of ALYREF at the mRNA and protein levels (Fig. 4E, G, H). It was also verified that, with ALYREF knockdown, although the mRNA expression level of MUC1 was not changed (Fig. 4F), the protein expression level was significantly downregulated (Fig. 4G, I). The results indicate that ALYREF is an important recognition protein for RNA m^5^C-methylated MUC1 mRNA.

### The RNA m^5^C reader ALYREF affects DR progression

**Fig. 5:**
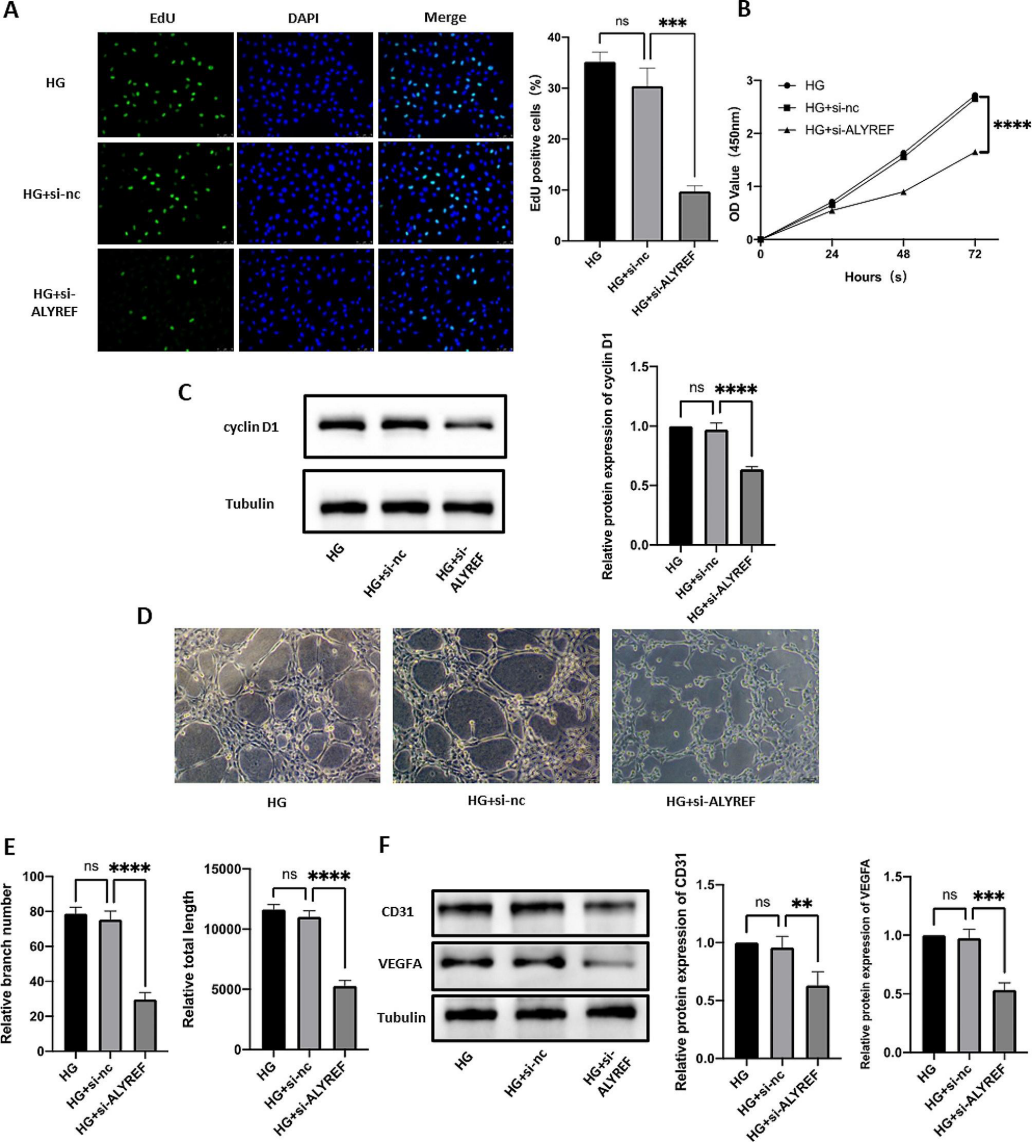
Effect of ALYREF on cell proliferation and angiogenesis. (**A**) An EdU assay was performed to detect the effect of si-ALYREF on cell proliferation. (**B**) A CCK-8 assay was performed to detect the effect of si-ALYREF on cell proliferation. (**C**) Western blot analysis of the changes in cyclin D1 protein levels after si-NSUN2 transfection. (**D, E**) Effect of HG and si-ALYREF on the tube formation ability of HRMECs was examined by tube formation assay. (**F**) Western blot was used to measure the changes in CD31 and VEGFA protein levels after si-ALYREF

**Fig. 6:**
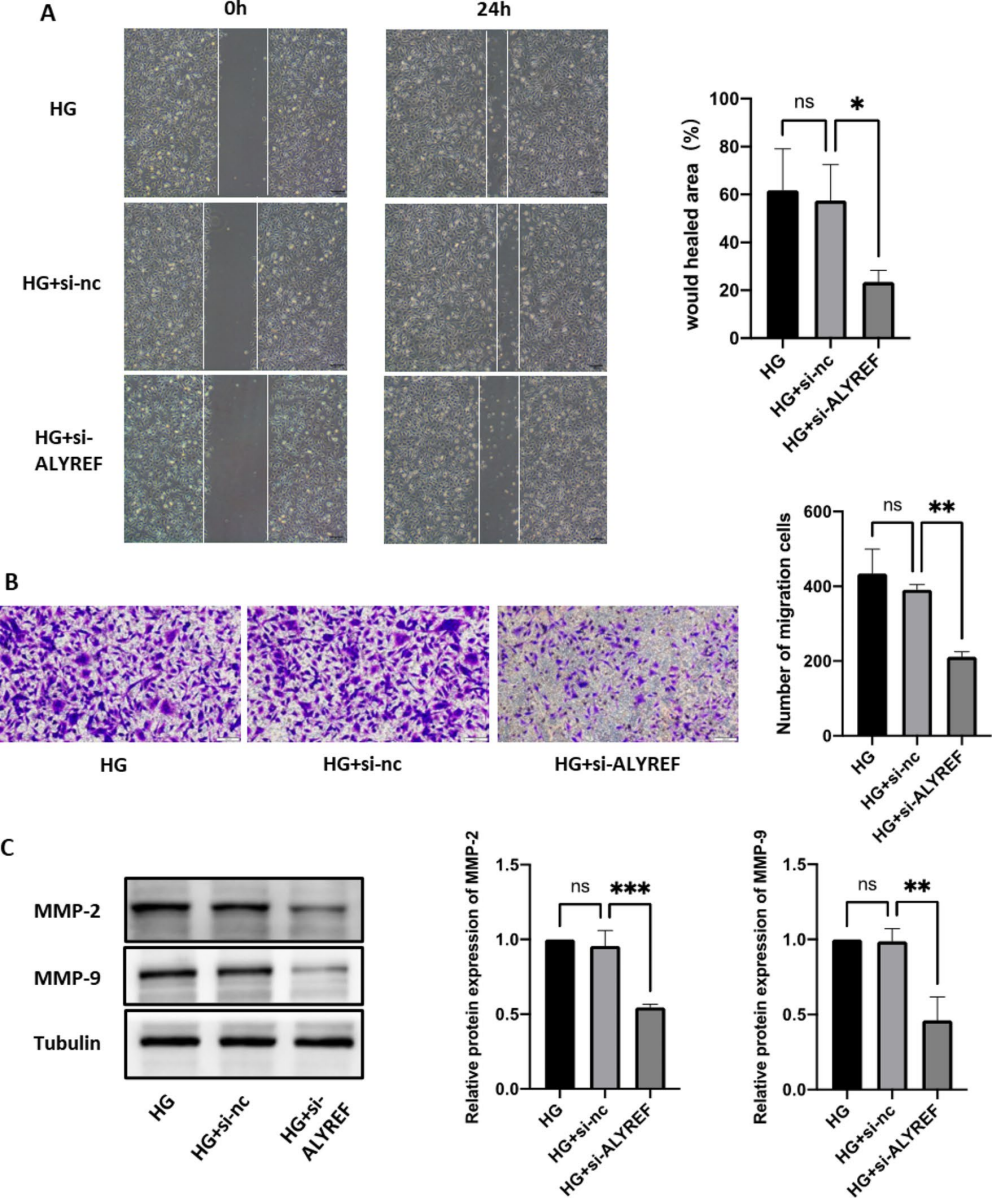
Effect of ALYREF on cell migration. (**A**) Wound healing assay was also conducted to evaluate the changes in cell migration ability after si-ALYREF. (**B**) A transwell assay was conducted to assess the changes in cell migration ability after si-ALYREF. (**C**) Western blot was used to examine the changes in MMP-2 and MMP-9 protein levels after si-ALYREF

Next, we further verified the role of ALYREF in DR progression. EdU and CCK8 results showed that cell proliferation was significantly reduced in the si-ALYREF group (Fig. 5A, B). Western blot analysis also confirmed that the cell proliferation-related protein cyclin D1 was downregulated in the si-ALYREF group (Fig. 5C). According to the tube formation assay, the tube formation ability of the si-ALYREF group was significantly lower than that of the control group (Fig. 5D, E), and the corresponding protein indicators CD31 and VEGFA also exhibited the same trend (Fig. 5F). Wound healing and Transwell assays showed that the migration ability of cells was reduced after ALYREF knockdown (Fig. 6A, B), and the migration-related proteins MMP-2 and MMP-9 also exhibited significant decreases (Fig. 6C).

### MUC1 is highly expressed in the DR model

**Fig. 7:**
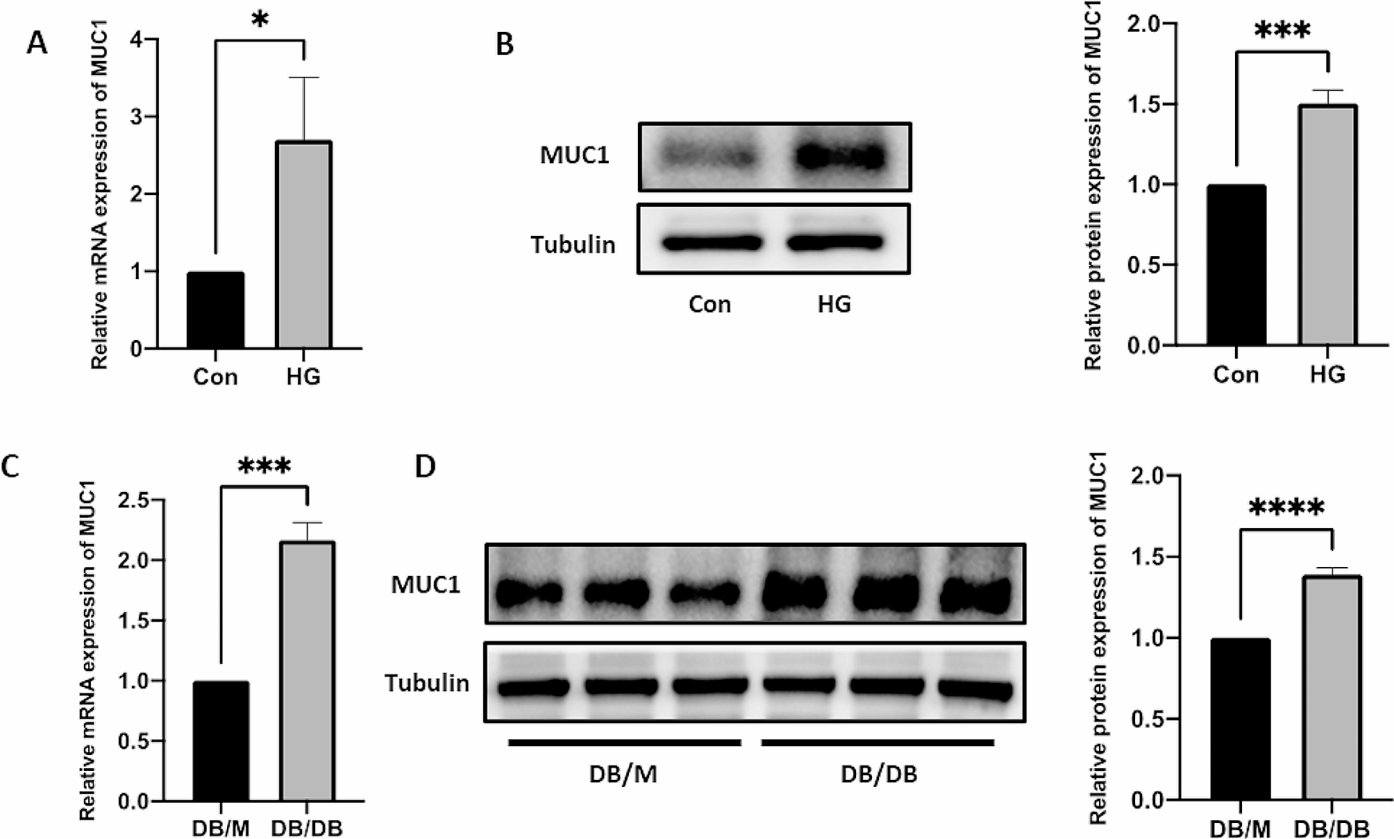
MUC1 is highly expressed in DR. (**A**) Differential expression of MUC1 mRNA expression levels at the cell level. (**B**) Differential expression of MUC1 protein expression levels at the cell level. (**C**) Differences in MUC1 mRNA expression levels in animal retinal tissues were measured via qRT-PCR. (**D**) Differences in MUC1 protein expression in animal retinal tissues were measured via Western blot

MUC1 is a transmembrane glycoprotein that can increase the expression levels of proangiogenic genes (such as CTGF, PDGF-B, and VEGF-A), thereby promoting angiogenesis and tube formation in endothelial cells [31]. Both qRT-PCR and Western blot verified that MUC1 was highly expressed in the DR cell model (Fig. 7A, B). Similarly, MUC1 mRNA and protein expression levels were increased in DB/DB mice (Fig. 7C, D). The results shown that the effect of knocking out MUC1 is similar to the effect of knocking out NSUN2(Additional file 1: Fig. S3, S4).

### Overexpressing MUC1 can rescue the progression of DR caused by NSUN2 silencing

**Fig. 8:**
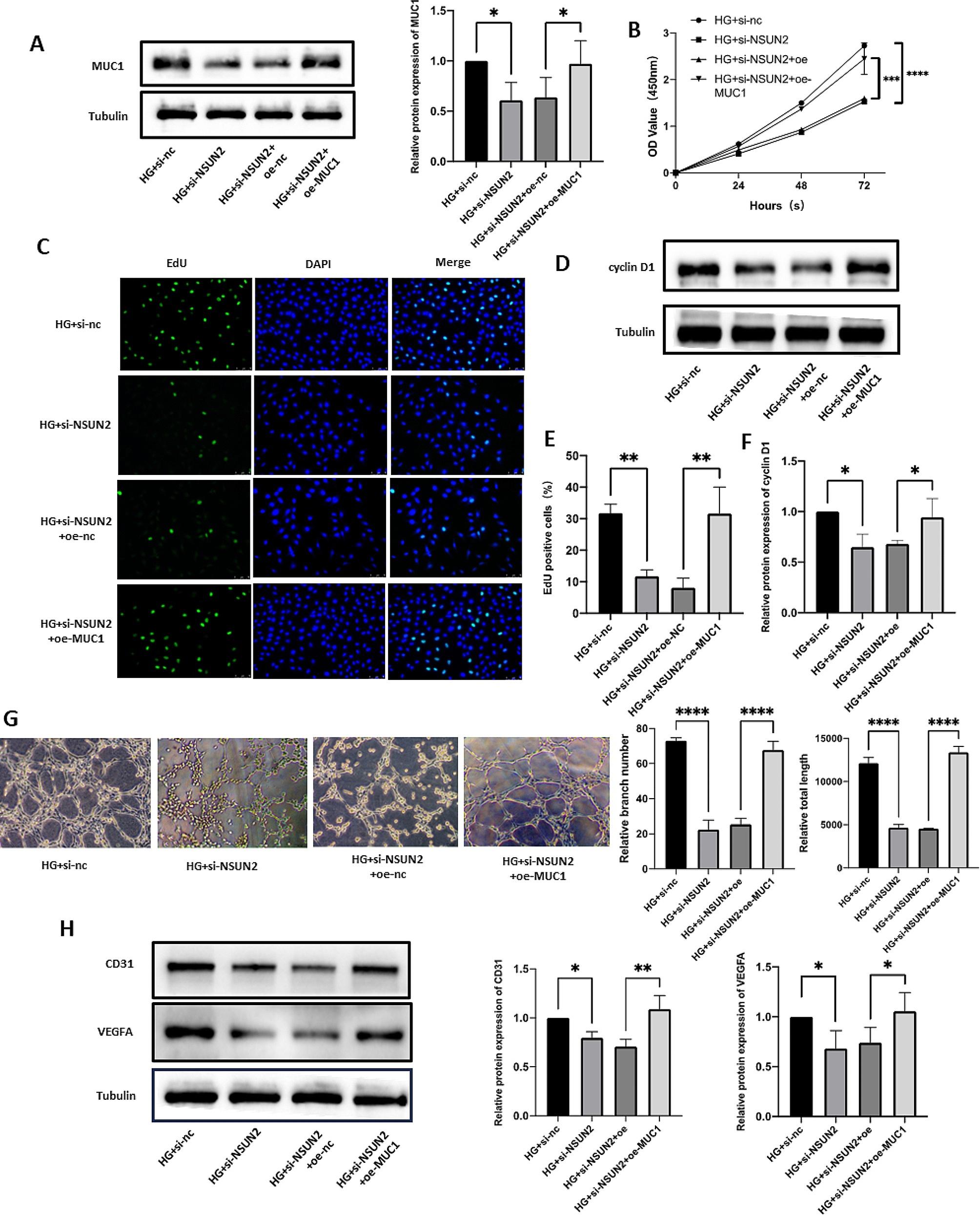
MUC1 partially rescued the effects of NSUN2 on proliferation and angiogenesis in HRMECs. (**A**) Western blot analysis of MUC1 protein levels after transfection with si-NSUN2 and/or oe-MUC1. (**B**) After si-NSUN2 and/or oe-MUC1 transfection, changes in cell proliferation were detected via an EdU incorporation assay. (**C, E**) After si-NSUN2 and/or oe-MUC1 transfection, changes in cell proliferation were detected via a CCK-8 assay. (**D, F**) Western blot was used to detect changes in cyclin D1 protein expression levels. (**G**) After si-NSUN2 or oe-MUC1 transfection, a tube formation assay was used to measure the changes in angiogenesis ability. (**H**) Western blot was conducted to assess CD31 and VEGFA protein expression levels

**Fig. 9:**
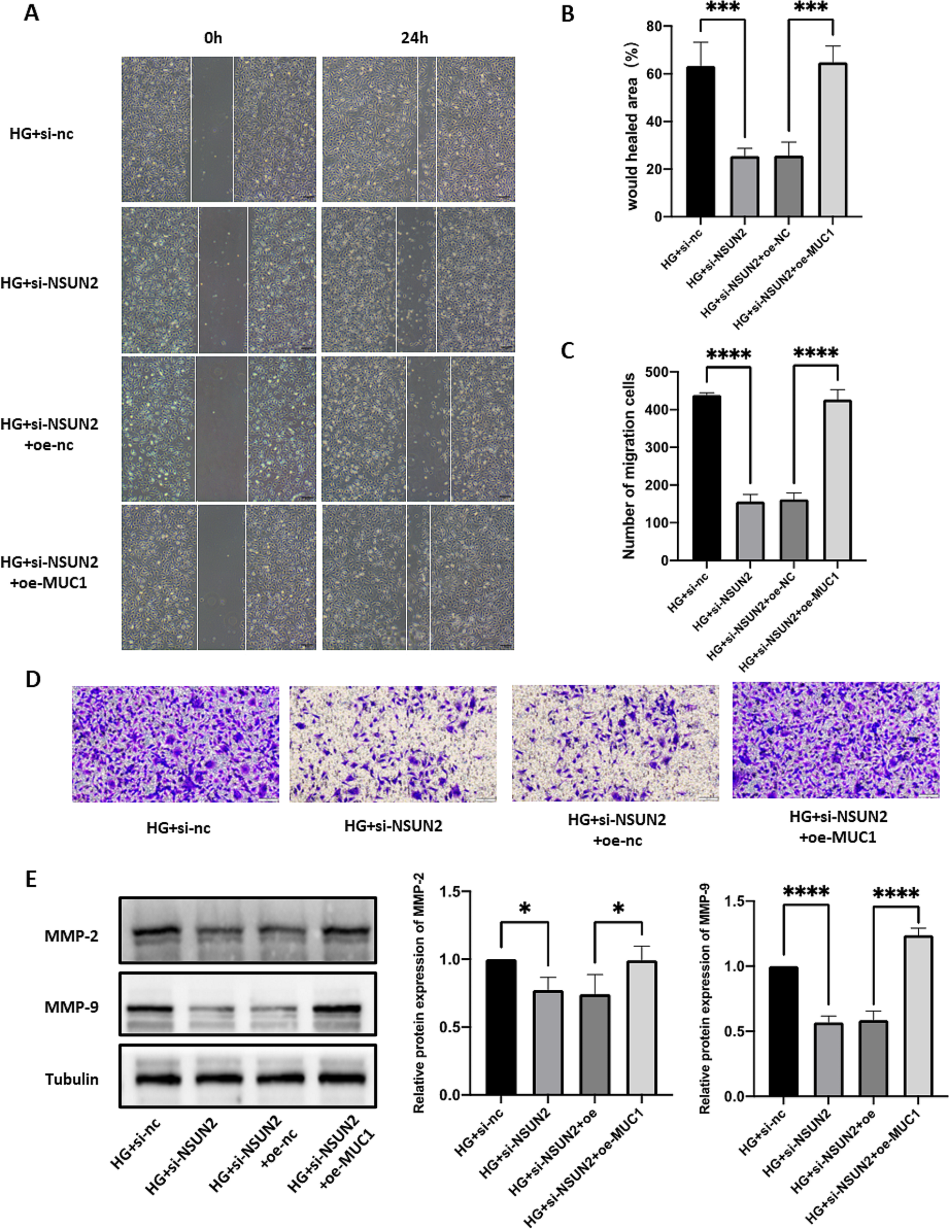
MUC1 partially rescued the effects of NSUN2 on the migration of HRMECs. (**A, B**) After si-NSUN2 and/or oe-MUC1 transfection, wound healing assays were performed to detect changes in cell migration ability. (**C, D**) After si-NSUN2 and/or oe-MUC1 transfection, a Transwell assay was used to evaluate the changes in cell migration ability. (**E**) Western blot was performed to measure the changes in the MMP-2 and MMP-9 protein expression levels after si-NSUN2 and/or oe-MUC1 treatment

We next examined whether MUC1 can improve the NSUN2-mediated DR process by overexpressing MUC1. Western blot results showed that overexpression of MUC1 could successfully reverse the decrease in MUC1 caused by NSUN2 knockdown (Fig. 8A). CCK8, EdU and Western blot assays showed that overexpression of MUC1 effectively improved the increase in cell proliferation caused by NSUN2 knockdown (Fig. 8B-F). In the tube formation assay, overexpression of MUC1 also effectively reversed the changes in tube formation caused by NSUN2 knockdown (Fig. 8G, H). Migration experiments also obtained the same results. Overexpressing MUC1 improved the decrease in the migration ability of HRMECs after si-NSUN2 transfection (Fig. 9A-E).

### Knockdown of NSUN2 in vivo significantly improved the progression of DR

**Fig. 10:**
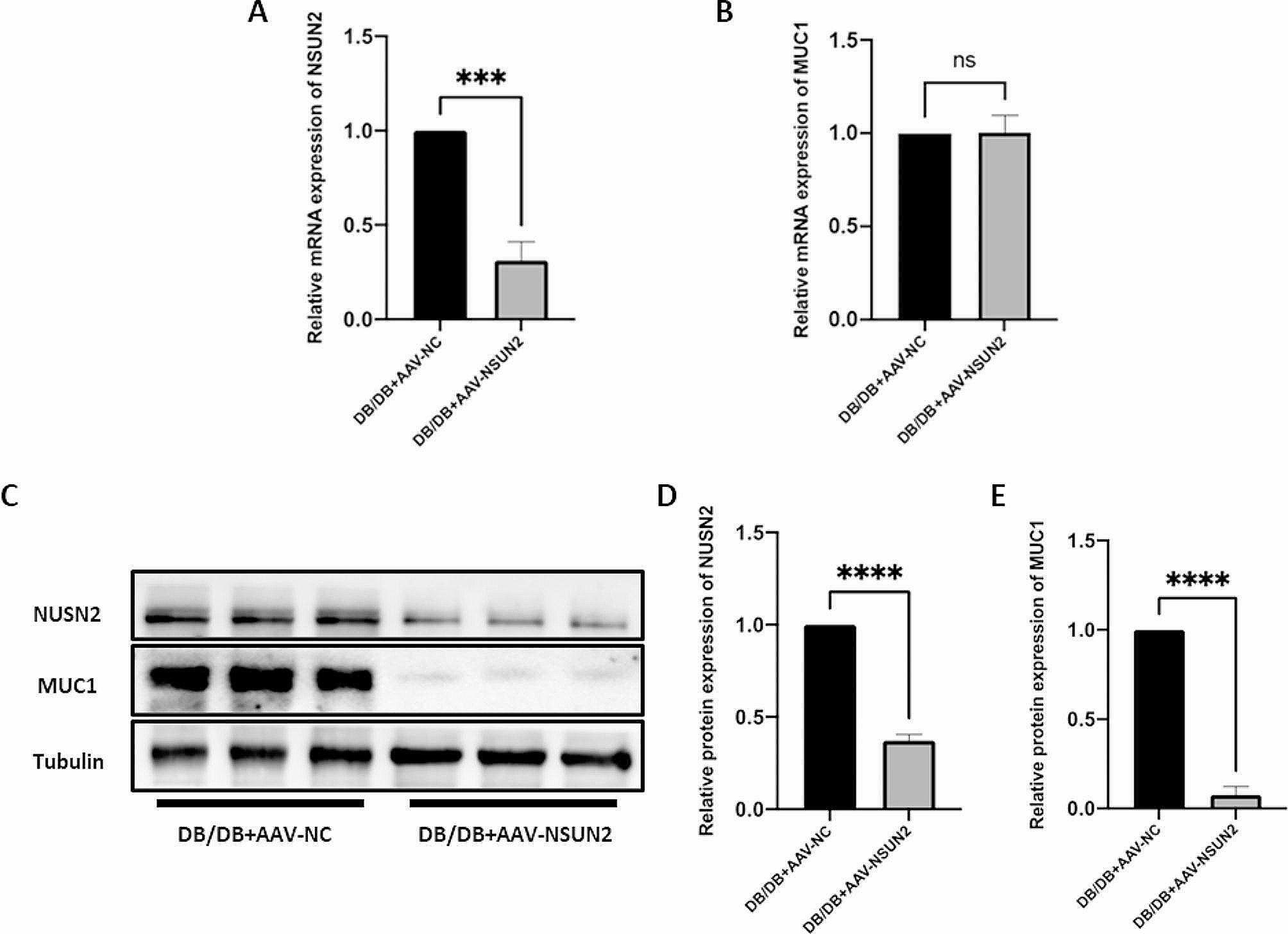
Transfection efficiency of NSUN2 in vivo and corresponding changes in MUC1. (**A, C, D**) The transfection efficiency of AAV-NSUN2 at the mRNA and protein levels. (**B**) Changes in MUC1 mRNA levels after NSUN2 knockdown in vivo. (**C, D**) Changes in MUC1 protein levels after NSUN2 knockdown in vivo. (**C, E**) Changes in MUC1 protein levels after NSUN2 knockdown in vivo

**Fig. 11:**
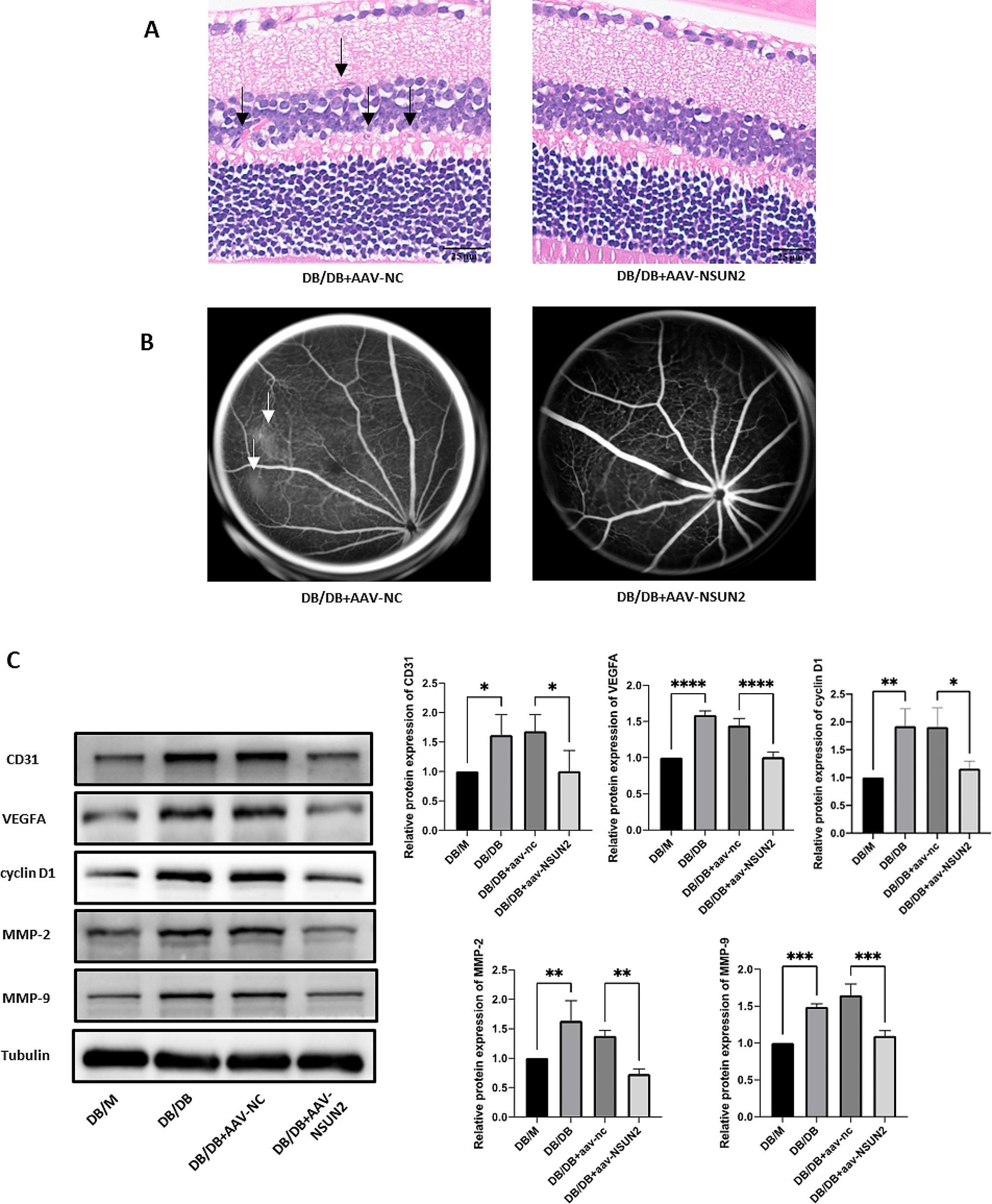
Corresponding changes in vivo after NSUN2 knockdown. (**A**) Representative images of HE staining after intravitreal injection of AAV-nc or AAV-NSUN2 in DB/DB mice. (**B**) Representative images of FFA after intravitreal injection of AAV-nc or AAV-NSUN2 in DB/DB mice. (**C**) After AAV-NSUN2 transfection, Western blot was used to detect CD31, VEGFA, cyclin D1, MMP-2, and MMP-9 protein expression

To further determine the impact of NSUN2 on DR progression, we used a mouse model of DR to further explore the role of NSUN2 in vivo. qRT-PCR and Western blot results confirmed that NSUN2 was successfully knocked down in vivo (Fig. 10A, C, D). Although the mRNA expression level of MUC1 was not altered by silencing NSUN2 (Fig. 10B), there was a significant reduction in the protein expression level (Fig. 10C, E). HE staining revealed retinal vascular proliferation in the inner nuclear layer of the retina, a loose arrangement of cells in the outer nuclear layer, and a sparse arrangement of ganglion cells with missing or blurred morphology in the DR mouse model group. After vitreous injection of AAV-NSUN2, these lesions were significantly improved (Fig. 11A). FFA assays revealed obvious microvascular leakage in the fundus of DB/DB mice, while no obvious leakage was found in the AAV-NSUN2 group (Fig. 11B). The retinal tissues of each group were removed for Western blot analysis. CD31, VEGFA, cyclin D1, MMP-2, and MMP-9 were highly expressed in the DR mouse model group, and their expression was significantly reduced after NSUN2 knockdown in vivo (Fig. 11C). Overall, these findings demonstrated that NSUN2 knockdown can effectively improve the progression of DR.

## Discussion

DR is the most common microvascular complication of diabetes and can cause retinal neovascularization by causing endothelial cell dysfunction, blood‒retinal barrier disruption, ischemia and hypoxia [32]. There is no good treatment for the early stage of DR. For the late stage of DR, anti-VEGF drugs, retinal laser photocoagulation and vitrectomy are commonly used in clinical treatment. However, the treatment efficacy is poor in some patients, and the side effects are severe [33]. Therefore, it is necessary to explore the molecular mechanisms regulating angiogenic factors to provide new strategies for the treatment and prevention of DR. Based on the results of the Microarray analysis, we selected NSUN2, which was significantly differentially expressed according to the Microarray analysis, as the research object. The NSUN family comprises RNA m^5^C methyltransferases that have been extensively studied in recent years. The most widely studied gene is NSUN2, which can increase the RNA m^5^C methylation level of a variety of RNAs, including tRNAs, mRNAs and noncoding RNAs [34, 35]. RNA m^5^C methylation is a widespread posttranscriptional modification that participates in a variety of biological processes by controlling RNA metabolism [36, 37]. Studies have shown that NSUN2 can increase the stability of LINC00324 by regulating m^5^C modification, thereby regulating glioma angiogenesis [38], and can also enhance the translation of intercellular adhesion molecule 1 (ICAM-1) to reduce inflammatory responses in the vascular endothelium [39]. However, the role of NSUN2-mediated m^5^C modification in DR is currently unclear.

In the present study, NSUN2 was found to be highly expressed in the vitreous fluid of DR patients, retinal tissue of DR model mice, and in a DR cell model, indicating that NSUN2 may be involved in the occurrence and development of DR. High RNA methylation levels were present in retinal tissues from both the DR mouse model and the DR cell model, suggesting that the progression of DR may be associated with RNA m^5^C methylation. DR is the most common microvascular complication caused by diabetes. The main pathological feature of this disease is that microangiogenesis causes blood vessel leakage, resulting in vitreous hemorrhage, and blood vessels pull on the vitreous surface, leading to retinal detachment [40, 41]. The results of this study confirmed that knockdown of NSUN2 can effectively inhibit the proliferation, invasion and formation of microvessels and can significantly inhibit the extent of RNA m^5^C methylation. In vivo experiments also confirmed that knockdown of NSUN2 reduced retinal damage and microangiogenesis in a mouse model of DR. Therefore, we can conclude that NSUN2 promotes the progression of DR.

Based on the review and integration of the literature, we propose that NSUN2 exerts a series of biological effects by affecting MUC1. MUC1 is a transmembrane mucin whose extracellular domain can serve as a ligand for stromal and endothelial cell adhesion receptors and participate in various functions, such as proliferation, migration, and invasion [42]. Studies have shown that MUC1 can induce angiogenesis by increasing the levels of neurosin-1 and its ligand VEGF [43]. Overexpressing MUC1 has been shown to promote angiogenesis by activating the Akt and ERK pathways and inducing multiple proangiogenic factors, including VEGF [44]. In the present study, the MeRIP-qPCR results showed that knockdown of NSUN2 could significantly inhibit the RNA m^5^C level of MUC1. After knockdown of NSUN2, although the mRNA level of MUC1 remained unchanged, the protein level was significantly reduced, suggesting that NSUN2 plays a key role in the translation of MUC1 in DR through posttranscriptional m^5^C modification. Subsequently, an MUC1 overexpression plasmid was constructed, and Western blot confirmed that MUC1 was overexpressed in HRMECs. Overexpressing MUC1 reversed the effects of NSUN2 knockdown on microvessel proliferation, invasion, and formation. In summary, this study confirmed that NSUN2 can affect MUC1 through RNA m^5^C methylation, thereby playing a role in the development of DR.

ALYREF and YBX1, as RNA m^5^C methylation-binding proteins, can promote the export and stability of mRNAs by recognizing RNA m^5^C methylation [45–47]. In the retinal tissue of a DR mouse model, ALYREF was highly expressed, while there was no significant difference in the expression of YBX1. Therefore, we explored the biological role of ALYREF and its impact on MUC1. After ALYREF was knocked down, the protein expression of MUC1 was significantly downregulated, and MUC1 significantly reduced the proliferation, invasion and angiogenesis of HRMECs. The above results showed that ALYREF is an important recognition regulator of MUC1 during the RNA m^5^C modification of DR.

**Fig. 12:**
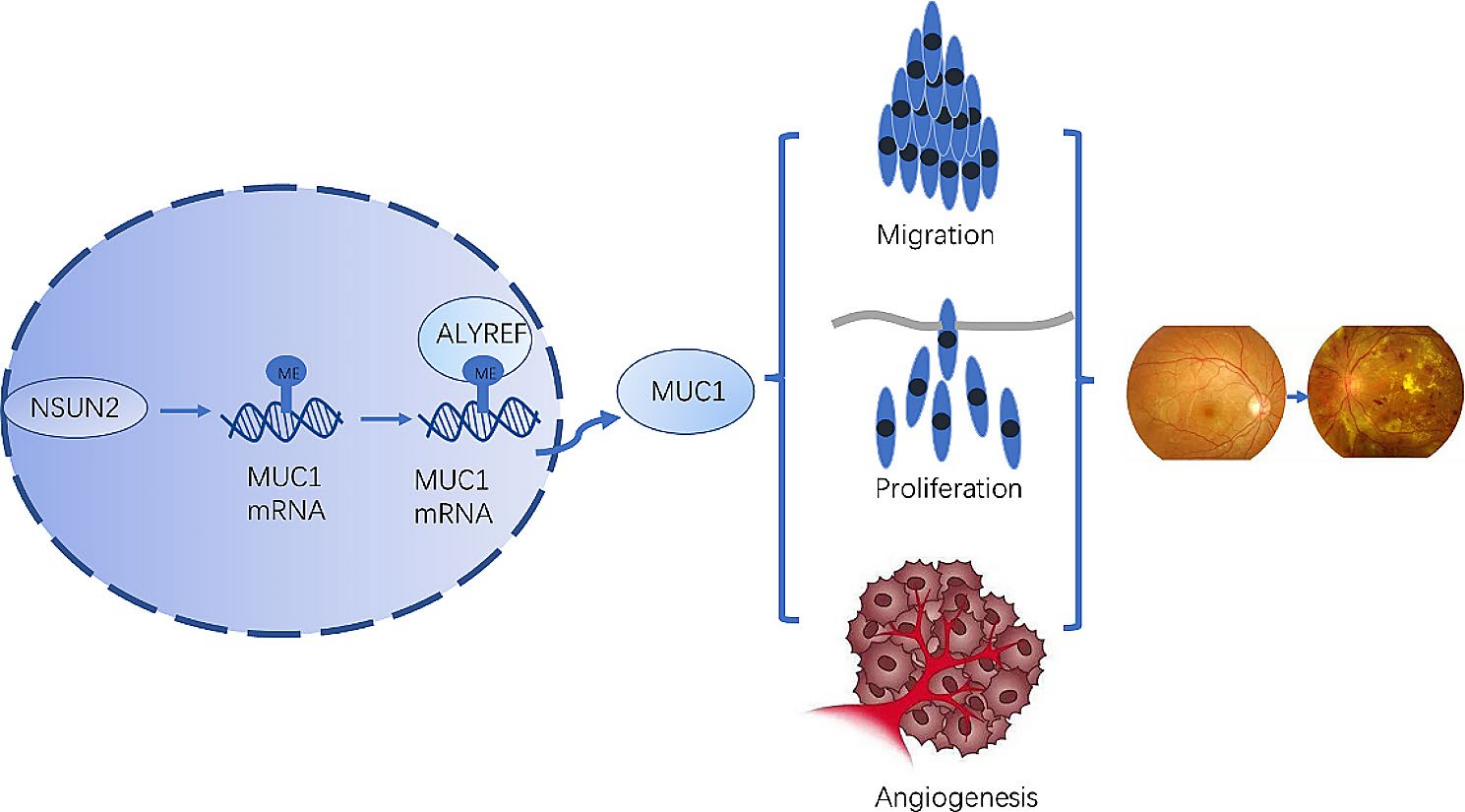
Potential mechanism through which NSUN2-mediated RNA m^5^C methylation regulates DR

In this study, we confirmed the pathological function, mechanism and downstream targets of NSUN2 in DR (Fig. 12). This is the first study on the function and regulatory mechanism of NSUN2 in DR. This article confirms the therapeutic potential of targeting NSUN2 in the treatment of DR and provides a new target for the future treatment of DR.

## Conclusions

NSUN2 leads to the activation of MUC1 through the RNA m^5^C methylation pathway by recognizing ALYREF. This activation affects microvascular proliferation, invasion, and angiogenesis in DR. The NSUN2/ALYREF/m^5^C-MUC1 signaling axis plays an important role in regulating DR and provides a new target for the future treatment of DR.

### Electronic supplementary material

Below is the link to the electronic supplementary material.

Supplementary Material 1

## Data Availability

All data generated for this study can be accessed from the corresponding author upon reasonable request.

## Abbreviations

DR: Diabetic retinopathy
HRMECs: Human retinal microvascular endothelial cells
m^6^A: N^6^-methyladenosine
m^1^A: N^1^-methyladenosine
m^7^G: N^7^-methyladenosine
m^5^C: 5-methylcytosine
NTD: N-terminaldomain
CTD: C-terminaldomain
CEWH: corneal epithelial wound healing
RB: Retinoblastoma
lipo: Lipofectamine
qRT-PCR: Quantitative real-time PCR
CCK-8: Cell Counting Kit-8
EdU: 5-Ethynyl-2′deoxyuridine
ECL: Chemiluminescence
MeRIP-qPCR: Methylated RNA immunoprecipitation-PCR
DAPI: 4,6-diamino-2-phenylindole
FFA: Fluorescein fundus angiography
HE: Hematoxylin and eosin
ANOVA: One-way analysis of variance
