# Supplementary material for: NSUN2 affects diabetic retinopathy progression by regulating MUC1 expression through RNA m5C methylation
Source: J Transl Med. 2024 May 19;22:476. doi: 10.1186/s12967-024-05287-4 (PMC11103891; doi:10.1186/s12967-024-05287-4)
Supplement: Supplementary file 1 — Supplementary Material 1 [file 12967_2024_5287_MOESM1_ESM.docx]

# Additional file 1

**
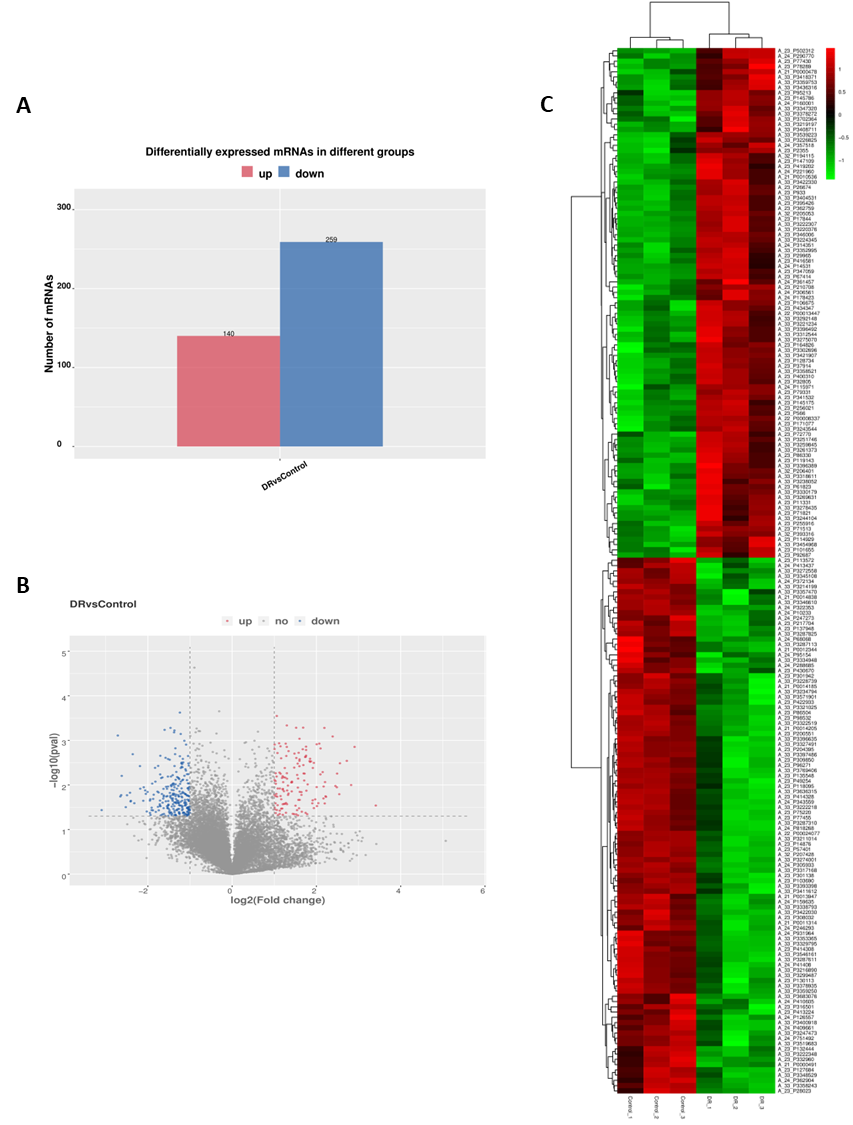
**

**Fig. S1: NSUN2 is highly expressed in Microarray analysis.**

**(A)** Column chart demonstrates the number of differentially expressed up- and down-regulated mRNAs. **(B)** Heatmaps demonstrates differentially expressed mRNAs between control group and DR group. **(C)** Volcano plot demonstrates differentially expressed mRNAs between control group and DR group.

**
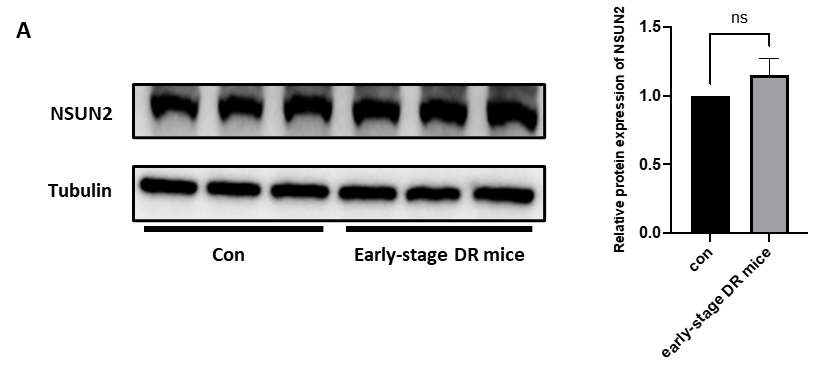
**

**Fig. S2: NSUN2 is not differentially expressed in early DR.**

**(A)** NSUN2 protein expression in control mice versus early DR mice.

**
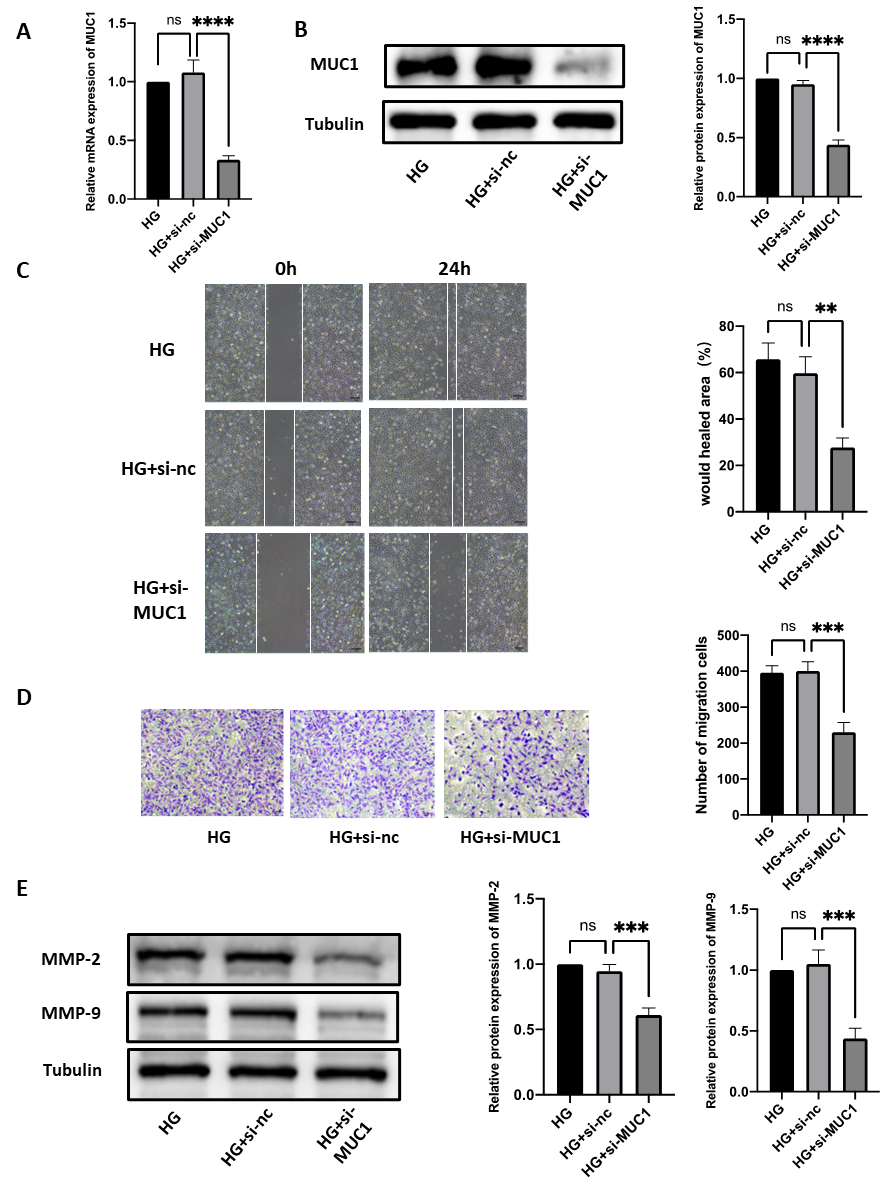
**

**Fig. S3: Effect of MUC1 on cell migration.**

**(A)** qRT-PCR was used to evaluate the transfection efficiency of MUC1. **(B)** Western blot analysis of the transfection efficiency of MUC1. **(C)** Wound healing assay was performed to detect changes in cell migration ability after si-MUC1 treatment. **(D)** Transwell assays were used to measure the alteration in cell migration ability after si-MUC1 transfection. **(E)** Western blot analysis of the changes in the MMP-2 and MMP-9 protein levels after si-MUC1 treatment.

**
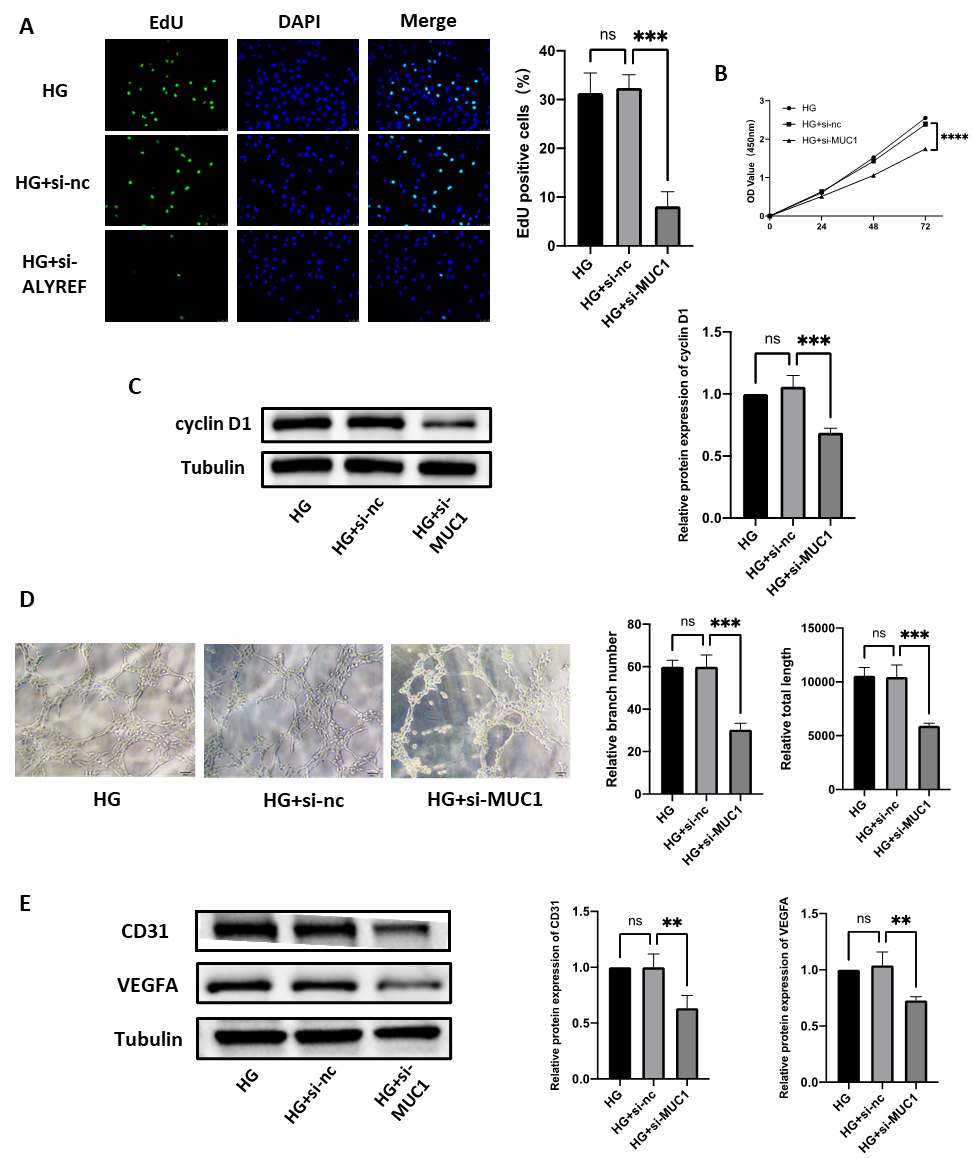
**

**Fig. S4: Effect of MUC1 on cell proliferation and angiogenesis.**

**(A)** An EdU assay was performed to detect the effect of si-MUC1 on cell proliferation. **(B)** A CCK-8 assay was performed to detect the effect of si-MUC1 on cell proliferation. **(C)** Western blot analysis of the changes in cyclin D1 protein levels after si- MUC1 transfection. **(D)** Effect of HG and si-MUC1 on the tube formation ability of HRMECs was examined by tube formation assay. **(E)** Western blot was used to measure the changes in CD31 and VEGFA protein levels after si-MUC1.

**Table S1: The sequences of siRNAs**

| siRNA | Sequence (5′-3′) |
| --- | --- |
| siNSUN2  siALYREF  siMUC1 | GCAGUGUCCCAUCGUCUUATT GGAAACTGCTGGTGTCCAATC  CGGGATACCTACCATCCTA |

**Table S2: Primer sequences for qRT-PCR**

| **Gene** | **Primer sequences** |
| --- | --- |
| NSUN2  （human）  NSUN2  （Mouse） | Forward: CAAGCTGTTCGAGCACTACTAC  Reverse: CTCCCTGAGAGCGTCCATGA  Forward: GGGAAAGCTGTCATCTGCTG  Reverse: CAGAGGCTTGGGTTTCCCTA |
| ALYREF | Forward: GCAGGCCAAAACAACTTCCC  Reverse: AGTTCCTGAATATCGGCGTCT |
| MUC1（human）  MUC1（Mouse）  Tubulin（human）  Tubulin  （Mouse） | Forward: CCTGCCTGAATCTGTTCTGC  Reverse: TGTAAGCACTGTGAGGAGCA  Forward: CGTCAGGCTCAGCTATCATTC  Reverse: GGGTATTGACTTGGCACTGAA  Forward: CAGCTCATCACAGGCAAGGA  Reverse: TGCTCTTACCAGCTTGCGAA  Forward:CAGCTCATCACAGGCAAGGA  Reverse:TGCTCTTACCAGCTTGCGAA |

**Table S3: Primary antibodies for Western blot assay**

| **Antibody name** | **Dilutions** | **Vendor name** | **Cat No.** |
| --- | --- | --- | --- |
| NSUN2 | 1:10000 | Proteintech | 20854-1-AP |
| ALYREF | 1:1000 | Abcam | ab202894 |
| YBX1 | 1:1000 | Abcam | ab76149 |
| MMP2 | 1:1000 | Proteintech | 10373-2-AP |
| MMP9 | 1:1000 | Proteintech | 10374-2-AP |
| CD31 | 1:10000 | Proteintech | 11265-1-AP |
| VEGFA | 1:1000 | Abcam | ab214424 |
| Cyclin D1 | 1:1000 | CST | 67116-1-Ig |
| MUC1 | 1:1000 | Abcam | ab45167 |
| Tubulin | 1:5000 | Proteintech | 66240-1-Ig |

**Table S4: The screened potential target genes of NSUN2/RNA m5C**

| **Gene sets** | **Gene numbers** | **Gene symbols** |
| --- | --- | --- |
| RNA m^5^C modified transcripts in Hela cells | 77 | GALM, WDR44, ACAA2, ROCK2, ECE1, PRDX5, FECH, LSP1, RIN2, PRDX6,DNAJC24, MTCH1, CAPNS1, QSOX1, HSPA9, TALDO1,TBL3, TMEM109, LMO7, GSTZ1, IST1, TPM2, HNRNPA1, EPHX1, SETD7, NOL9, RCN2, ETFB, CISD1, TPM1, MAPK3, FLNB, AUH, RABIF, GCLC, TKT, ECI1, NAV2, ADPRHL2,PEBP1, CBX8, SND1,TMEM33,ARHGAP27, MIEN1, EFHD2, TPT1, RPS28, LUZP1, CTSC, RPS9, POLDIP2, LTIH4, SHMT2, UBAC1, CCDC51, CPOX, UHRF1, RYR1, CNN3, GPX1, PDLIM7, NOLC1, DUT, MGAT2, SSR3, UTP6, COPG1, HINT1, MIF, DDX21, FOCAD, MUC1, RRM2, IMP4, DDX18, TPM4 |
| Upregulated proteins | 39 | RYR1, LSP1,TPM4, NOLC1,TPM1,**MUC1**,SHMT2, TPM2, TBL3, CTSC, DDX21, RRM2, MGAT2, LTIH4, EFHD2, RPS28,IMP4, HSPA9, ECE1, DDX18, FLNB, DUT, TMEM33, LUZP1, RPS9, SSR3, PDLIM7, NOL9, TPT1, CPOX, SND1, QSOX1, HNRNPA1, COPG1, CNN3, POLDIP2, UTP6, UHRF1, CCDC51 |
